# Marine heatwave decimates fire coral populations in the Caribbean

**DOI:** 10.1073/pnas.2518506122

**Published:** 2025-11-17

**Authors:** Emilia C. Dell’Antonio, Lauren Mahoney, Peter J. Edmunds

**Affiliations:** ^a^Department of Biology, California State University, Northridge, CA 91330-8302

**Keywords:** coral reefs, ecology, *Millepora*, mass mortality

## Abstract

Marine heatwaves (MHW) are common destructive events affecting coral reefs. After decades of degradation, the shallow reefs of the United States Virgin Islands have been depleted of scleractinian corals, leaving abundant colonies of the hydrozoan fire coral *Millepora* dominating the coral community. This dominance ended in 2024 after 84% of *Millepora* colonies over 43 km of shore were killed by a MHW that brought the hottest October in the 36 y since monitoring began. In August 2024, dead *Millepora* were rare on these reefs, but by March 2025, severe bleaching created a fire coral graveyard. Decimation of the fire coral biotope shows that these short-term coral winners are unlikely to be future reef builders.

Marine heatwaves (MHW) are thermal anomalies exceeding long-term average seawater temperatures and are occurring with increased frequency and intensity (1, 2). These extreme elevations in temperature place organisms at the upper limits of their thermal tolerance, causing mass mortalities (2, 3). The effects of MHW are acute on tropical reefs where they cause scleractinian corals to bleach and die, profoundly altering community structure and function (4).

*Millepora* are calcareous hydrocorals that are common on tropical coral reefs where they share many features with scleractinians, including functioning as reef builders (5, 6). Known as “fire corals” due to their potent nematocysts (6), in the Caribbean and tropical west Atlantic, most *Millepora* exploit a sheet-tree morphology (5–7). This strategy allows this coral to preempt space on hard surfaces with encrusting sheets and to take advantage of flow and particulates in the water column with branches (5–7). Branches create structural refuges for invertebrates and juvenile fishes (8, 9), providing an important community function as structure-forming scleractinians have declined in population size (10). As Caribbean coral reefs have degraded, *Millepora* has flourished (10), in part because their colonies often recover from bleaching, although some large-scale mortalities have occurred (6, 11, 12). In the US Virgin Islands, long-term studies of coral reefs (13) have documented the death of scleractinians, the increased abundance of macroalgae, and the rise in ecological importance of *Millepora* (10, 14). By August 2024, *Millepora* appeared to be the only coral flourishing on these shallow reefs, but 7 mo later, their populations were decimated.

## Results

**Fig. 1.**
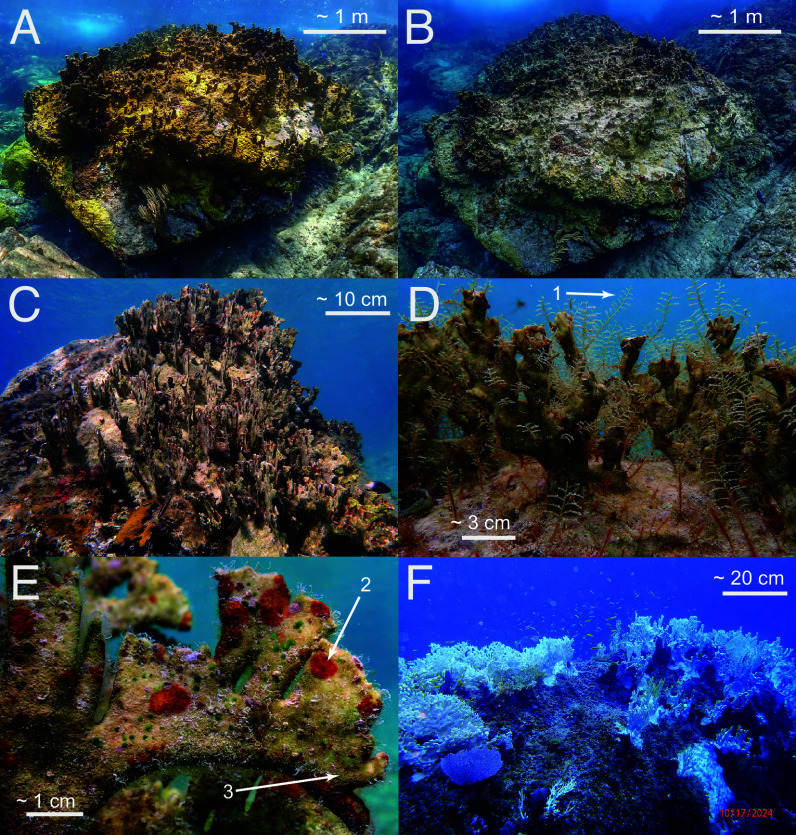
Dead *Millepora* colonies overgrown by algae and invertebrates, compared to healthy and bleached colonies. The transition from (*A*) live *Millepora* in 2024 to (*B*) dead colonies in 2025. (*C*–*E*) Dead-in-place branches of *Millepora* overgrown by hydroids (*Sertularella* spp., arrow 1) and PAC (arrow 2), note gastropores and dactylopores (arrow 3) where polyps occurred. (*F*) Bleached *Millepora* colonies at ~5 m depth taken at Kiddel Bay (~900 m east of Cabritte Horn). Photo credit (*A* and *B*): P.J. Edmunds; (*C*–*E*): E.C. Dell’Antonio; (*F*): J. Miller. Scale bars approximate.

**Fig. 2.**
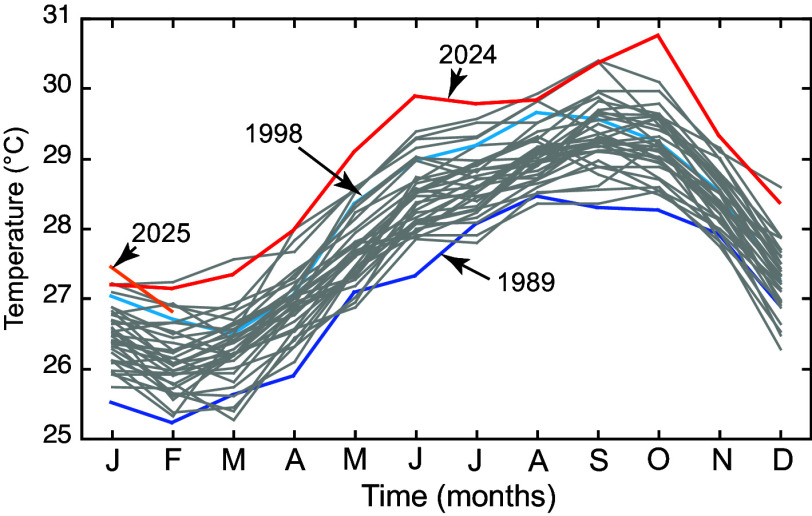
Mean monthly seawater temperature at Yawzi Point (9 m depth). Gray lines show the means for the last 35 y, while the red line shows the 2024 MHW, orange 2025, dark blue 1989, and light blue shows the 1998 El Niño. J = January, F = February, etc.

**Fig. 3.**
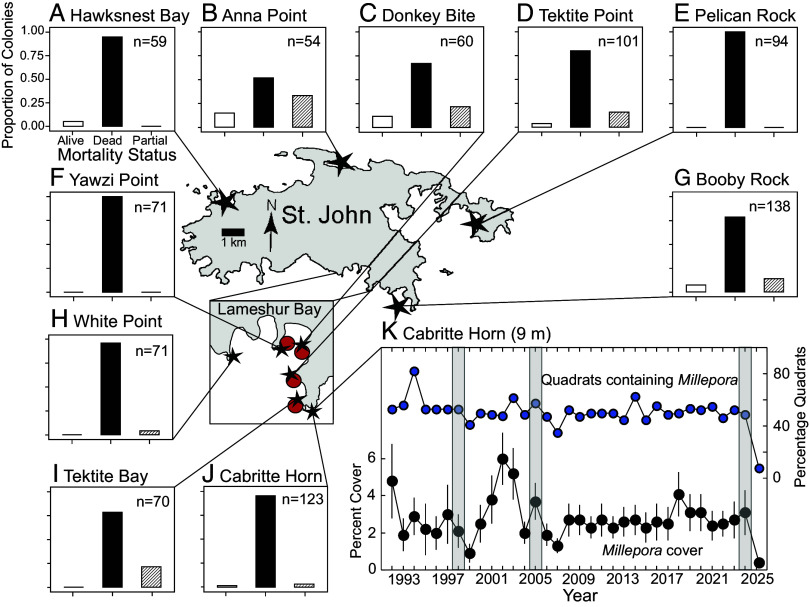
Study sites and *Millepora* abundances in St. John. Locations of mortality surveys are indicated by black stars and invertebrate surveys by red circles. (*A*–*J*) Proportion of colonies that were alive (white), dead (black), and with partial mortality (diagonally striped) at each site in March 2025 (n, number of colonies; common axes labeled in *A*). (*K*) Abundance of *Millepora* at Cabritte Horn (9 m depth) from 1992 to 2025. Percentage cover (mean ± SE, left *Y* axis) and percentage of quadrats containing *Millepora* (right *Y* axis); n = 16 to 17 to 1999, and 30 to 44 thereafter. Gray bars show bleaching events.

In August 2024, mean *Millepora* cover on hard substrata at <10 m depth in Lameshur Bay, St. John, was 12.1 ± 2.4% (mean ± SE, n = 6 sites). By March 2025, *Millepora* mortality around St. John was severe, leaving colonies dead-in-place (Fig. 1 *A*–*C*). Dead *Millepora* colonies were colonized by ephemeral hydroids and early-successional algae including peyssonnelioid algal crusts (PAC) and turfs, among which dactylopores and gastropores were visible where *Millepora* polyps formerly occurred (Fig. 1 *D* and *E*). This suggests that the corals died a few months earlier, coincident with the hottest October recorded in 36 y (Fig. 2) when severe bleaching affected *Millepora* (Fig. 1*F*). Seawater temperatures were ≥0.73 °C above the 35-y mean every month in 2024, with the anomaly peaking in October at +1.55 °C. Mean monthly temperatures from June to December 2023 were ≥0.45 °C hotter than the long-term average, exacerbating the effects of the 2024 MHW that followed. Photographs highlight the transition from golden brown living colonies of *Millepora* in 2024 (Fig. 1*A*), to drab and dead colonies in March 2025 (Fig. 1*B*), illustrating the death of 86.1 ± 0.1% (mean ± SE, n = 6 sites) of colonies between White Point and Cabritte Horn (Fig. 3).

At Cabritte Horn, the mean cover of *Millepora* at 9 m depth declined by 87% (Mann–Whitney *U* test, U = 1,098.50, n_1_ = 41, n_2_ = 38, *P* < 0.001) from 2024 to 2025, with a mean cover of 2.8 ± 0.2% (±SE) over 33 y (1992 to 2024) but only 0.4 ± 0.3% (±SE, n = 38 quadrats) in March 2025 (Fig. 3*K*). Over four decades, *Millepora* was recorded in 52% of the photoquadrats annually surveyed at Cabritte Horn (mean = 34/y), but in March 2025, it was found in only 8% of the photoquadrats (n = 38). The low cover of *Millepora* at Cabritte Horn in March 2025 (0.4%), and the small proportion of colonies categorized as alive (4%) or partially alive (11%) (Fig. 3*J*), demonstrates the extent to which the population was reduced. High mortality extended along 43 km of the shore, with 51 to 100% (84.2 ± 0.1%, mean ± SE) of *Millepora* dead at 10 sites <5 m in depth (n = 54 to 138 colonies/site), with mortality reduced at north shore compared to south shore sites (Chi-Square, χ^2^ = 8.235, df = 1, *P* = 0.004).

## Discussion

*Millepora* bleaching has been recorded on Caribbean reefs since the 1980s (6), but the 2024 MHW and its effects in St. John were unprecedented relative to previous events. Bleaching occurred on reefs that have endured decades of disturbances, depressing coral cover at ≤9 m depth to ∼2% by 2024 (13). *Millepora* had high relative importance in St. John, occupying nearly half as much benthos as scleractinians (pooled among taxa) on average over 33 y (Dataset S2). While *Millepora* cover previously dropped at Cabritte Horn following bleaching in 1998 and 2005 (Fig. 3*K*), it remained ≥0.9% (i.e., >56% higher than in 2025) following these events, and small colonies were still found in 35 to 41% of the photoquadrats (Fig. 3*K*). Further, the persistent low cover of scleractinians (i.e., <10%) suggests that previous postbleaching recovery of *Millepora* (i.e., after 1998 and 2005) did not reflect the occupancy of space that was ceded by scleractinians during the same bleaching events. The 2024 MHW was also unprecedented in severity and duration (Fig. 2), and its arrival following the 2023 MHW appears to have overwhelmed the ability of *Millepora* to recover from high temperature and bleaching.

Despite the susceptibility of *Millepora* to bleaching, colonial modular taxa with sheet-tree morphologies [i.e., *M. complanata* and *M. alcicornis* (5, 6)] have features favoring persistence through disturbances, including elevated temperature and storms (7). At Cabritte Horn, *Millepora* abundances from 1992 to 2021 demonstrate how the proliferation of branches during calm periods and their loss during storms is traded against the spread of sheets during benign conditions, and their shrinkage during MHWs when synergy with downwelling light promotes bleaching and death (7). The trade-off between sheet and tree morphologies in Caribbean *Millepora* enhances ecological persistence in the genus, which has led to the development of a *Millepora* biotope in St. John striking in its ecological resilience relative to scleractinians.

**Fig. 4.**
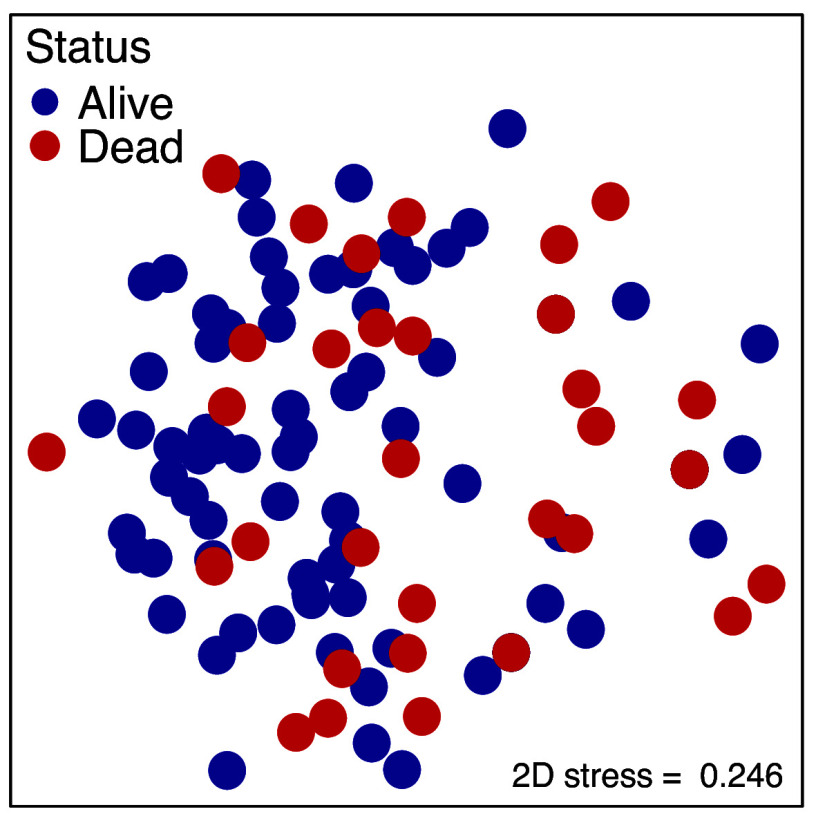
Multivariate invertebrate community structure on alive (in August 2024) and dead (in March 2025) colonies of *Millepora* at <5 m depth in St. John. Assemblages described in 2-dimensional ordination space using non-metric multidimensional scaling (NMDS) with log-transformed presence/absence data and resemblance matrix prepared using Jaccard dissimilarities.

By March 2025, this biotope was wiped out to create a horizontally stratified graveyard of fire coral that is likely to have ecological impacts on reef-wide community dynamics. When *Millepora* dies, its ecological functions are weakened, including the ability to produce carbonate skeletons that stabilize mobile substrata, deter vacant space occupancy by macroalgae, and form a 3-dimensional habitat through new branches (5–7). However, invertebrate assemblages associated with *Millepora* did not change from August 2024 to March 2025 (Fig. 4), suggesting that dead-in-place colonies continue to provide ecological services through structural refugia (6), at least for ~5 mo. Nevertheless, the algal taxa now growing on their skeletons (e.g., PAC) are likely to intensify the algal-dominated community state and may initiate a change in the invertebrates with which the dead colonies are associated, thus creating a potential extinction debt (15). The few remaining relic colonies of *Millepora*, mostly located in deeper water, shaded microhabitats, or on sides of branches where reduced light exposure may have protected them from bleaching (7), are likely to contribute to future population recovery. Such an outcome, however, is likely to take years, if not decades, to occur due to the sparse number of relic colonies (0.5 ± 0.2 colonies/10 m^2^, mean ± SE, n = 10 sites), the negative genetic implications of a highly clonal population structure, and short dispersal distances of pelagic propagules in this genus (6).

## Materials and Methods

Seawater temperature was recorded at 9 m depth at Yawzi Point using Hobo loggers (U22-001, ±0.2 °C) at 0.001 Hz and averaged by day from 1989 to 7/2022, and a Seabird logger (SBE39+, ±0.002 °C) from 8/2022 to 3/2025. Coral cover (including *Millepora*) has been recorded annually since 1992 at Cabritte Horn (and five other sites between and White Point) using photoquadrats randomly placed along a fixed transect. *Millepora* mortality was quantified in March 2025 as the proportion of dead, partially dead, and alive colonies using 200+ m^2^ band transects at 1- to 3-m depth at 10 sites around St. John (Fig. 3). At four sites in Lameshur Bay, invertebrate communities associated with *Millepora* before and after the mass mortality event were recorded along randomly placed transects.

Detailed methods, data, and supporting statistical analyses are available in *SI Appendix*.

## Supplementary Material

Appendix 01 (PDF)

Dataset S01 (CSV)

Dataset S02 (CSV)

Dataset S03 (CSV)

Dataset S04 (CSV)

Dataset S05 (CSV)

Dataset S06 (CSV)

Dataset S07 (CSV)

## Data Availability

All study data are included in the article and/or supporting information.
