# Supplementary material for: Marine heatwave decimates fire coral populations in the Caribbean
Source: Proc Natl Acad Sci U S A. 2025 Nov 17;122(47):e2518506122. doi: 10.1073/pnas.2518506122 (PMC12663973; doi:10.1073/pnas.2518506122)
Supplement: Supplementary file 1 — Appendix 01 (PDF) [file pnas.2518506122.sapp.pdf]

Supporting Information for

Marine heatwave decimates fire coral populations in the Caribbean

Emilia C. Dell'Antonio[1], Lauren Mahoney[1], Peter J. Edmunds[1]

[1]Department of Biology, California State University, Northridge, CA 91330-8302, USA.

Corresponding Author: Peter J. Edmunds

Email: peter.edmunds@csun.edu

This PDF file includes:

Supporting text (Materials and Methods)

Legends for Datasets S1 to S7

SI References

Other supporting materials for this manuscript include the following:

Datasets S1 to S7

Supporting Information Text Materials and Methods

Seawater temperature was recorded at 9-m depth at Yawzi Point using mostly Hobo loggers (U22-001, ± 0.2°C) at 0.001 Hz and averaged by day from 1989 to 2022 (Data S1). From August 2022, a Sea-Bird logger (± 0.002°C, SBE 39plus) was deployed with a sampling interval of 0.001 Hz, and records were averaged by day.

Coral cover has been recorded annually at six sites between Cabritte Horn and White Point using photoquadrats (Cabritte Horn reported in Data S2) (1). These sites were randomly selected in 1992 and include Cabritte Horn for which *Millepora* cover is reported here for 1992 to 2025 (Fig. 3) (Data S3). From 1992 to 1999, transects were 20 m long and 20 photoquadrats were recorded, and from 2000 to present, transects were extended to 40 m, and 40 photoquadrats were annually recorded; all photoquadrats (0.5 × 0.5 m) were recorded at random positions along one fixed transect at each site. Photoquadrats were recorded with color slide film from 1987 to 2000 in a Nikonos V camera fitted with two strobes and mounted on a rigid framer, and from 2001, digitally with a variety of cameras with resolution extending from 3.34 megapixels to 45.7 megapixels as camera technology advanced. The rigid framer held the cameras perpendicular to the reef to reduce the effects of parallax. For overall community structure, the pictures were analyzed using either CPCe (2) or CoralNet (3) software with 200 dots randomly located on each image and manually annotated for benthic substratum groups.

Millepora percent cover was recorded at Cabritte Horn from 1992 to March 2025 (Data S3). Sampling in March 2025 was opportunistic and completed with a handheld camera (12 megapixels), held above a quadrat (0.5 × 0.5 m). Color slides were digitized (4000 dpi) and images were analyzed in Image J software (4), using the freehand selection tool to outline the encrusting bases of all *Millepora* colonies and calculate their area (cm^2); dead enclosed portions of living colonies were subtracted from the outlined area. The areas of colonies were summed by photoquadrat and expressed as percentage cover for graphical display. Proportional cover of *Millepora* was compared among sampling years between 1992 and 2005 using a zero-inflated beta generalized linear mixed model (GLMM) with quadrat included as a random effect. This analysis did not reveal a difference in *Millepora* cover over 34 years (Chi-square = 38.27, df = 33, P = 0.242). A contrast of *Millepora* percentage cover between 7/2024 and 3/2025 revealed a significant reduction in association with the MHW of 2024 (Mann Whitney U test, U = 1098.50, n_1 = 41, n_2 = 38, P < 0.001). The GLMM was completed using the package *glmmTMB* (5) in R (R version 2025.05.1), and the Mann Whitney test was completed using Systat 13 software.

In July 2024, the percent cover of *Millepora* at <10-m depth was recorded at six additional locations that are near, but do not overlap the long-term monitoring sites in Great Lameshur Bay

(Data S4). To quantify *Millepora* abundance on hard substrata across the depth profile of the reef, quadrats (0.25 m^2) partitioned into 25 sub-squares were placed every 0.6 m of depth along four replicate transects oriented perpendicular to isobaths at each site. The transects extended from the surface to the depth at which sand became the dominant substratum (between 5- and 7-m depth). The number of squares in which *Millepora* was the dominant substratum in each quadrat were counted to calculate percent cover of *Millepora* with 4% resolution. For the present analysis, quadrats were pooled among depths at each site to quantify the abundance of *Millepora* at <10m depth across Great Lameshur Bay.

To assess the extent of *Millepora* mortality around St. John, corals was evaluated on scuba in March 2025 by counting the number of dead *Millepora* colonies in 200 m^2 plots along isobaths that were 1 to 3 m in depth at six sites that were near but did not overlap the long-term monitoring sites in Great Lameshur Bay (Fig. 3) (Data S5). Study areas were haphazardly selected at each site and dead *Millepora* colonies were counted ± 1 m of a 100-m transect. *Millepora* mortality was also evaluated on snorkel at three additional sites on the northern shore and eastern side of St. John to extend the spatial scale of analysis. In these surveys, the number of dead colonies was counted along isobaths that were 1 to 3 m in depth using band transects (2 m wide) covering areas ranging from 200 to 576 m^2 (Data S5). Survey locations at the three sites on the northern shore and eastern side of St. John were haphazardly selected, and the transects were marked at the beginning and end using a handheld GPS (Garmin 78) with a 2-m width maintained using plastic poles (Data S5). The length of each survey plot was determined as the track length between GPS points in Google Earth (version 7.3.6.10201).

To determine whether *Millepora* mortality was variable around St. John, colonies of *Millepora* at all sites were scored based on their status: “Dead”, when they were devoid of *Millepora* tissue and showed signs of recent death (Fig. 1A, B); “Alive”, when they had no signs of recent tissue death and healthy *Millepora* tissue was present; and “Partially Alive”, when they had signs of recent death and some live *Millepora* tissue. Since the MHW-mediated mortality occurred about five months before these surveys, the margins of the dead colonies were readily discernible, allowing ‘colony’ (categorized as Alive, Dead, or Partially Alive) to be used as the statistical replicate. *Millepora* mortality (number of colonies Dead vs (Alive + Partially Alive)) was compared among locations around St. John with a contingency table (table S1) and a Chi-Square test. For this analysis, sites were pooled to support a contrast of the South Shore (six sites between Cabritte Horn and White Point + Booby Rock) and North Shore (Hawksnest Bay + Anna Point); locations shown in (Fig. 3). This analysis revealed a significant difference in *Millepora* mortality among shores (Chi-Square Test, $!^2 = 8.235$, df = 1, $P = 0.004$), with less mortality than expected by chance alone on the North Shore. Chi-Square analysis was performed using the package *stats*

(6) in R (R version 2025.05.1).

To determine whether *Millepora* death affected the invertebrates with which they were associated when alive, invertebrate communities associated with *Millepora* were quantified in July/August 2024 and March 2025 at Cabritte Horn, Donkey Bite, Yawzi Point, and Tektite Point (Data S6). Quadrats (0.25 m^2, n = 10) were placed every meter along 10-m transects along a narrow band between 1- to 4-m depth at each site. In 2024, all invertebrates on *Millepora* tissue within these quadrats were enumerated and in 2025, all invertebrates on dead *Millepora* colonies that were inferred to have been killed by the 2024 MHW were enumerated. Invertebrates were identified using reference photographs and a field guide (7) (Data S7). Data were collapsed to a nominal scale (i.e., present or absent) so that the analysis could include the ephemeral hydroid, *Sertularella* spp. that was found on dead colonies in 2025 but was not encountered in 2024. *Sertularella* spp. was scored after the surveys were completed using photographs of each quadrat recorded in 2024 and 2025 in which this hydroid was scored as present or absent. Differences in invertebrate communities associated with *Millepora* before and after colony death were visualized using two-dimensional non-metric multidimensional scaling (NMDS) prepared using a resemblance matrix constructed using Jaccard dissimilarity (*vegan* package in R version 2025.05.1 (8)).

Data S1. (separate file). Long-term temperature data from St. John, US Virgin Islands. Data collected from January 1989 to March 2025. These data are displayed in Fig. 2

Data S2. (separate file). Percentage cover of scleractinian coral at 9-m depth at Cabritte Horn from 1992 to 2024.

Data S3. (separate file). Percentage cover of *Millepora* at 9-m depth at Cabritte Horn from 1992 to 2025. These data are displayed in Fig. 3J as mean (± SE) percent cover together with the number of photoquadrats sampled each year that contained *Millepora*.

Data S4. (separate file). *Millepora* percent cover at <10-m depth in August 2024 at six sites between White Point and Cabritte Horn [Cabritte Horn, Donkey Bite, Little Lameshur Bay, Tektite Point, Yawzi Point, White Point].

Data S5. (separate file). *Millepora* colonies categorized as Alive, Dead, or Partial Alive in surveys at ten sites around St. John conducted in March 2025. Sites were pooled into two categories to support a contingency table analysis of mortality (Dead versus (Alive + Partially Alive)) along the South shore [Cabritte Horn + Donkey Bite + Tektite Bay + Tektite Point + Yawzi Point + White Point + Booby Rock] and North shore [Hawksnest Bay + Anna Point]) (Fig. 3).

Data S6. (separate file). Presence (1) or absence (0) of invertebrates on live (in 2024) and dead (in 2025) *Millepora* colonies at four sites between White Point and Cabritte Horn [Cabritte Horn, Donkey Bite, Tektite Point, Yawzi Point (Fig. 3)]. These data are displayed in Fig. 4 as an NMDS using Jaccard dissimilarity.

Data S7. (separate file). Species list of conspicuous invertebrates found on live (in 2024) and dead *Millepora* colonies (in 2025).

SI References

1. P.J. Edmunds, The hidden dynamics of low coral cover communities. *Hydrobiologia* **818**, 193-209 (2018) doi:10.1007/s10750-018-3609-9
2. K. E. Kohler, S. M. Gill, Coral Point Count with Excel extensions (CPCe): A Visual Basic program for the determination of coral and substrate coverage using random point count methodology. *Computers & Geosciences* **32**, 1259-1269 (2006). doi:10.1016/j.cageo.2005.11.009
3. O. Beijbom *et al.*, Towards Automated Annotation of Benthic Survey Images: Variability of Human Experts and Operational Modes of Automation. *PLOSOne* **10**, 1-22 (2015). doi:10.1371/journal.pone.0130312
4. M. D. Abramoff, P. J. Magelhaes, S. J. Ram, Image Processing with ImageJ. *Biophotonics International* **11**, 36-42 (2004).
5. M. E. Brooks *et al.*, glmmTMB Balances Speed and Flexibility Among Packages for Zeroinflated Generalized Linear Mixed Modeling. *The R Journal* **9**, 378-400 (2017). doi: 10.32614/RJ-2017-066.
6. R Core Team, R: A Language and Environment for Statistical Computing. Foundation for Statistical Computing, Vienna, Austria, (2025). https://www.R-project.org/.
7. P. Humann, N. DeLoach, L. Wilk, Reef Creature Identification: Florida Caribbean Bahamas (New World Publications Inc., ed. 3, 2013).
8. J. Oksanen *et al.*, vegan: Community Ecology Package, R package version 2.6-4 (2022); https://CRAN.R-project.org/package=vegan
